# Whole-Cell Properties of Cerebellar Nuclei Neurons *In Vivo*

**DOI:** 10.1371/journal.pone.0165887

**Published:** 2016-11-16

**Authors:** Cathrin B. Canto, Laurens Witter, Chris I. De Zeeuw

**Affiliations:** 1 Netherlands Institute for Neuroscience, Royal Dutch Academy of Arts & Sciences, Amsterdam, The Netherlands; 2 Department of Neuroscience, Erasmus Medical Center, Rotterdam, The Netherlands; University Paris 6, FRANCE

## Abstract

Cerebellar nuclei neurons integrate sensorimotor information and form the final output of the cerebellum, projecting to premotor brainstem targets. This implies that, in contrast to specialized neurons and interneurons in cortical regions, neurons within the nuclei encode and integrate complex information that is most likely reflected in a large variation of intrinsic membrane properties and integrative capacities of individual neurons. Yet, whether this large variation in properties is reflected in a heterogeneous physiological cell population of cerebellar nuclei neurons with well or poorly defined cell types remains to be determined. Indeed, the cell electrophysiological properties of cerebellar nuclei neurons have been identified *in vitro* in young rodents, but whether these properties are similar to the *in vivo* adult situation has not been shown. In this comprehensive study we present and compare the *in vivo* properties of 144 cerebellar nuclei neurons in adult ketamine-xylazine anesthetized mice. We found regularly firing (N = 88) and spontaneously bursting (N = 56) neurons. Membrane-resistance, capacitance, spike half-width and firing frequency all widely varied as a continuum, ranging from 9.63 to 3352.1 MΩ, from 6.7 to 772.57 pF, from 0.178 to 1.98 ms, and from 0 to 176.6 Hz, respectively. At the same time, several of these parameters were correlated with each other. Capacitance decreased with membrane resistance (R^2^ = 0.12, P<0.001), intensity of rebound spiking increased with membrane resistance (for 100 ms duration R^2^ = 0.1503, P = 0.0011), membrane resistance decreased with membrane time constant (R^2^ = 0.045, P = 0.031) and increased with spike half-width (R^2^ = 0.023, P<0.001), while capacitance increased with firing frequency (R^2^ = 0.29, P<0.001). However, classes of neuron subtypes could not be identified using merely k-clustering of their intrinsic firing properties and/or integrative properties following activation of their Purkinje cell input. Instead, using whole-cell parameters in combination with morphological criteria revealed by intracellular labelling with Neurobiotin (N = 18) allowed for electrophysiological identification of larger (29.3–50 μm soma diameter) and smaller (< 21.2 μm) cerebellar nuclei neurons with significant differences in membrane properties. Larger cells had a lower membrane resistance and a shorter spike, with a tendency for higher capacitance. Thus, in general cerebellar nuclei neurons appear to offer a rich and wide continuum of physiological properties that stand in contrast to neurons in most cortical regions such as those of the cerebral and cerebellar cortex, in which different classes of neurons operate in a narrower territory of electrophysiological parameter space. The current dataset will help computational modelers of the cerebellar nuclei to update and improve their cerebellar motor learning and performance models by incorporating the large variation of the *in vivo* properties of cerebellar nuclei neurons. The cellular complexity of cerebellar nuclei neurons may endow the nuclei to perform the intricate computations required for sensorimotor coordination.

## Introduction

The cerebellum is involved in preparation, coordination and timing of movements. During sensorimotor control cerebellar nuclei neurons (CNNs) integrate excitatory inputs mediated by mossy fiber and climbing fiber collaterals with inhibitory input from Purkinje cells (PCs), which form the sole output of the cerebellar cortex [1–6]. CNNs in turn project to various regions inside and outside the olivocerebellar system [7–12]. Glutamatergic CNNs project to different midbrain structures such as the red nucleus, reticular formation or thalamus and/or they project back to the cerebellar cortex [7,8,10,13–15]. Analogously, glycinergic inhibitory CNNs can also project to the brainstem and provide feedback to the cerebellar cortex, but with different termination patterns [11,12,16,17]. Most gamma-aminobutyric acid (GABA)-ergic projection neurons of the cerebellar nuclei (CN) provide an inhibitory projection to the inferior olive to regulate climbing fiber activity [18–20], while other GABAergic neurons may function as local interneurons [21]. This implies that the different types of neurons encode and integrate complex information that is likely reflected in a large variation of intrinsic membrane properties and integrative capacities of individual neurons. In addition to their complex afferent and efferent connectivity, CNNs also have diverse neurotransmitter and messenger RNA expression patterns [22]. Whether this large variation in connectivity patterns and cell properties will be reflected in a heterogeneous physiological cell population with well-defined physiological cell types needs to be determined. Up to now, the cell physiological properties of CNNs have been identified in young animals *in vitro* [12,17,23–28], but not in adults *in vivo* [29–31]. One may expect the variation of cell physiological properties *in vivo* to be larger compared to the *in vitro* situation [32], but this remains to be shown for the CNNs. Moreover, one may expect that it is hard to characterize the cellular properties of different types of neurons of a relatively homogenous subcortical structure like the CN with merely cell physiological parameters. In this respect, they stand in marked contrast to those of cortical structures, where neurons can often be characterized well by both unique axonal and dendritic trees and related succinct physiological properties [33–39]. Indeed, the cyto-architecture and related cell physiological properties of a neuron largely determine its specific computational capabilities [40–47]. Thus to prepare a solid ground for realistic modeling of CNNs we set out to do a systematic and comprehensive survey examining all types of CNNs *in vivo* in P21-42 old mice using standardized and blind conditions. We deem this information to be particularly relevant for large scale computational modeling of the olivocerebellar system [40–43], linking the cellular responses of CNNs to sensorimotor and behavioral parameters [44–47]. We describe whole-cell activity of more than hundred CNNs *in vivo* distributed across the different CN with most of our recordings being performed in the nucleus interpositus. In a subset of those CNNs we describe the response characteristics following optogenetic PC stimulation and in another subset we provide the main morphological characteristics for verification. We conclude that CNNs form a heterogeneous group with a substantial variation of cell physiological and morphological parameters covering large ranges of values. These parameters are continuously distributed, highlighting the rich repertoire of CNNs required for sensorimotor control.

## Methods

### Ethical statement and conflicts of interest

All procedures were approved by the animal committee of the Royal Dutch Academy of Arts & Sciences (DEC-KNAW). All procedures adhered to the European guideline for the care and use of laboratory animals (Council Directive 86/6009/EEC). All authors declare no financial or non-financial conflict of interest.

### Surgery

Eighty-nine C57BL/6 wild-type mice (21–42 days) and ten L7-ChR2(H134R)-eYFP mice (28–42 days; L7 promotor = PC Protein 2 (PCP2) promotor) [48–50] were prepared for whole-cell recordings in vivo by placing a custom made pedestal on the skull. In short, animals were anesthetized using isoflurane (5% induction, 1.5% in 0.5 L/min O_2_ and 0.2 L/min air), while body temperature was kept constant at 37°C via a feedback-controlled heating pad. The skin was shaved and incised for approximately 1 cm mid-sagittal on the skull. The bone was etched (37.5% phosphoric acid, Kerr) and a primer (Optibond, Kerr) was applied before a pedestal containing two M1.4 nuts was glued to the skull using dental acrylic (Flowline, Hereaus Kulzer). Animals received pain relief in the form of 2 mg/kg metacam (AUV). Animals were allowed to recover for at least one day before recordings were performed.

### Electrophysiology

On the day of the experiment the mouse was anesthetized with an intra-peritoneal injection of 75 mg/kg ketamine and 12 mg/kg xylazine. During the experiment the level of anesthesia was checked by monitoring of the heartbeat, whisker movements and reflexes. Anesthesia was supplemented when needed during the experiment. The occipital bone was exposed by removing the skin and neck muscles. A craniotomy covering nearly the entire occipital bone was made to allow access to the brain. Patch pipettes were pulled from borosilicate glass (Hilgenberg or Harvard Apparatus, 1.5mm OD, 0.86 ID) with a resistance of 4 to 9 MΩ and filled with (in mM): 10 KOH, 3.48 MgCl_2_, 4 NaCl, 129 K-Gluconate, 10 hepes, 17.5 glucose 4 Na_2_ATP, and 0.4 Na_3_GTP (295–305 mOsm; pH 7.2). The electrode was quickly lowered to depths between 1400–1500 μm with high pressure on the electrode. ‘Neuron hunting’ was done by making 2 μm steps under low (15 to 20 mbar) pressure up to a depth of 2400 μm. The total distance the electrode traveled from the surface of the brain was noted for all recordings (track-length). Seal resistance of all recordings was above 1 GΩ and recordings were amplified using a Multiclamp 700B amplifier (Axon Instruments) and digitized between 10 and 50 Khz using a Digidata 1440 (Axon Instruments). Bridge balance and capacitance neutralization were employed for all recordings. Junction potential between the electrode and extracellular milieu was determined to be -8.53 mV ± 0.87 mV. Before going to whole-cell mode, the pipette capacitance was neutralized in voltage clamp. In some neurons, up to a few minutes of spontaneous activity was recorded in voltage clamp before breaking in. In whole-cell mode, a 10 mV pulse was applied and the resulting current response was recorded. Input and access resistance were estimated by applying 10 mV square pulses of 100 ms duration in voltage clamp, small enough not to activate voltage-gated conductances. Membrane input and access resistance were calculated from an average of at least 5 responses. With pipette capacitance compensated, we used the initial response to determine access resistance (Ra), the relaxation to steady-state to determine τ, and the steady-state to determine the membrane resistance (Rm) (in combination with Ra). With these three parameters known, membrane capacitance (Cm) could be estimated. All other parameters were calculated from current clamp recordings. Firing frequency was estimated from spontaneous activity monitored immediately after switching to current clamp. To measure the firing frequency, we recorded for at least 15 seconds under baseline conditions. To quantify the regularity of firing of a neuron we used the coefficient of variance (CV) [CV = standard deviation (SD) (all inter-spike-intervals (ISIs))/mean (all ISIs)] and for the regularity of firing on small timescales we used the CV_2_ measure [CV_2_ = 2 | ISIn+1 –ISIn | / (ISIn + ISIn+1)]. Furthermore, we checked for depolarizing after-potentials (DAP). The fast after-hyperpolarization peak (fAHP) amplitude was calculated for the peak aligned spikes and defined by the difference in amplitude between the action potential voltage firing threshold and the maximum voltage deflection immediately following a spike. The time point of the fAHP was defined as the difference in time between the maximum voltage deflection following a spike and the time point of the peak of the spike. In response to 100 pA depolarizing current pulses we calculated, the spike amplitude and voltage firing threshold as well as maximal firing frequency. Negative current injection was used to quantify rebound firing of action potentials. Current steps were also used to quantify spike-frequency accommodation.

### Inclusion and exclusion criteria for neurons

All included CNNs had a seal resistance of at least 1 GΩ. Moreover, a bridge balance and capacitance neutralization were consistently employed. An online quality check was performed immediately by applying ten 10 mV steps to a neuron, while the neuron was kept at a holding potential of -60 to -65 mV. This allowed an immediate estimation of the access resistance. If access resistance was higher than 100 MΩ the neuron was excluded. Neurons were excluded when the baseline was drifting, when the seal or access resistance was bad or if one of the compensations was not performed.

### Optogenetic stimulation

Timed stimulation of channelrhodopsin was performed with a light-emitted diode (LED) driver capable of driving three LEDs at a maximum of five watts of power per LED [37]. Light intensity was set for the latter with a ten-turn dial. Three LED lights (465 nm, 60 lm, LZ1-B200, LED Engin, San Jose, California), positioned around the cerebellum of the mouse, were used to illuminate the whole cerebellum. This stimulus was powerful enough to activate PCs on every trial [37].

### Histology

Part of the cells was filled with 0.5% Neurobiotin (Vector labs). The neurons were filled throughout the whole recording for at least 20 minutes. In general, this resulted in a complete labelling of the recorded neuron (see results section for details). To control for potential impact of histological procedures we processed the brains according to two different protocols. In both cases staining procedures were only executed following successful retraction of the electrode from the cell, as indicated by an outside-out patch. For the first subset of staining experiments (N = 11 mice) the animals were perfused with 0.5% paraformaldehyde and 2.5% glutaraldehyde in 0.11 M phosphate buffer (PB) containing 4% sucrose. The brain was post-fixed for one night at 4°C in the same solution, after which it was cut into 100 μm thin slices and stained with avidin-biotin complex (Vector labs) followed by 3,3′-Diaminobenzidine (DAB) staining. Neurons labelled with ABC-DAB were visualized using a normal transmission-light microscope. For the second subset of experiments (N = 7 mice) the animals were decapitated after the experiment and brain slices were cut in ice-cold oxygenated “slicing” solution containing (in mM): 2.5 KCl, 1 CaCl2, 3 MgCl2, 25 NaHCO3, 1.25 NaH2PO4, 240 sucrose, 25 D-glucose and 0.01 kyneurenic acid. Subsequently, the slices were post-fixed in 0.2 M PB containing 4% paraformaldehyde and stored at 4°C for at least 24 hours. The slices were washed overnight in 0.2 M PB buffer and the next day they were washed in phosphate-triton buffer (PB-TX) (1% Triton in 0.2 M PB) followed by incubation in 5% goat serum (Sigma-Aldrich) in PB-TX for 2–3 hours on a rotator. Afterwards the slices were stained with streptavidin conjugated to Alexa 568 (Life technologies; 1:300) in PB-TX. Slices were again washed in PB and eventually dehydrated through increased concentration of alcohol and cleared in methyl salicylate [51]. Neurons were then reconstructed using computer-assisted Neurolucida (MicroBrightField) and a neuron specific shrinkage factor was applied to all reconstructions. The shrinkage factor was calculated by comparing the shrinkage of the processed brain slice with that of a living and freshly sliced unprocessed brain slice.

### Data analysis and statistics

Analysis was performed using Clampfit (Axon Instruments, version 10.2), Matlab 2010b (The Mathworks) and Excel (Microsoft). For statistics, we used unpaired t-tests in Excel or in Matlab, unless indicated otherwise. Regression analysis was conducted in Excel. Analysis of variance and clustering was conducted in Matlab. K-means clustering was done with Matlab’s built in functions. Variance explained for a certain clustering was determined by dividing the between groups variance by the total variance as assessed by an F-test. The optimal number of clusters was determined by the ‘elbow method’, where the optimal number of clusters is determined as the point where adding additional clusters does not significantly increase the amount of variance explained. Reported numbers in the text are mean ± SD, unless noted otherwise.

## Results

### Clustering of cerebellar nuclei neurons based on intrinsic electrophysiological parameters

**Fig 1:**
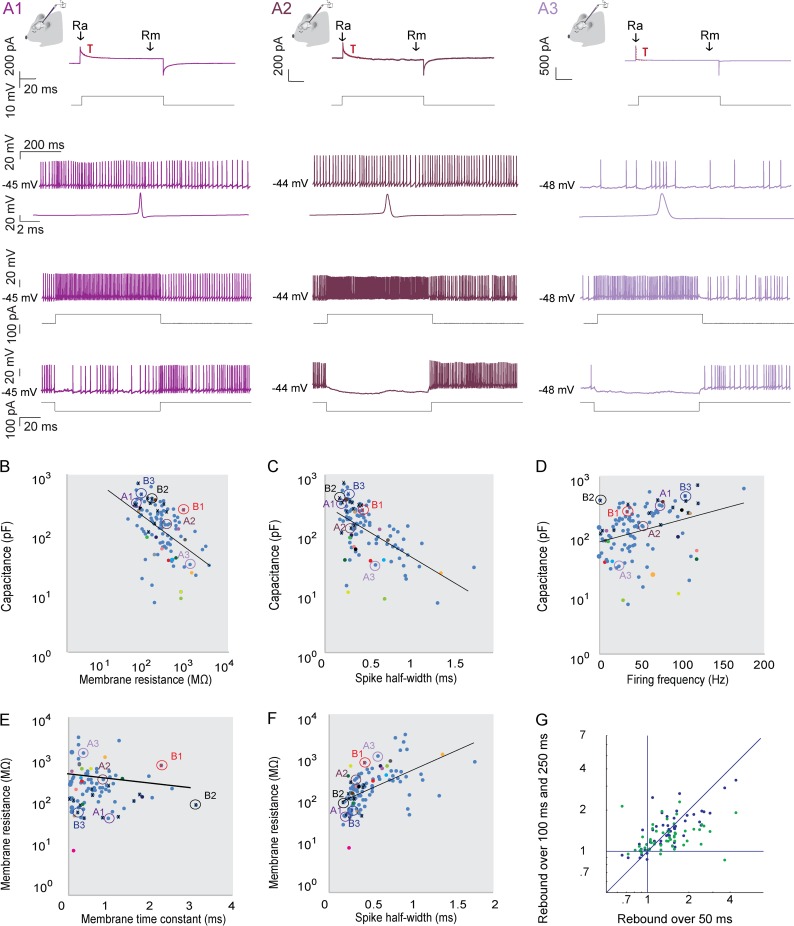
Intrinsic properties of CNNs. (A) Three representative CNNs recorded from *in vivo* in anesthetized mice. Top two traces: Voltage clamp recordings of the three representative CNNs *in vivo* in whole-cell mode. Estimations for access resistance and membrane resistance were obtained by applying a square 10 mV voltage pulse (lower black trace) to the neuron. Top trace: Current trace for each example neuron with the time point for measuring the access resistance (Ra), membrane resistance (Rm) and membrane time constant (τ) indicated. Third trace: Spontaneous activity of a CNN in current clamp. Neurons in the CN often fire spontaneously with varying firing frequencies. Some neurons are regular firing neurons (A1 and A2), others fire in bursts (A3). Fourth trace: Peak-aligned averages of spikes. Current response (fifth graph) in response to a 100 pA square pulse (sixth black trace). Bottom traces: Current response to a - 100pA step. CNNs can show a strong hyperpolarization-activated depolarizing current, which can lead to a rebound burst of action potentials. (B-G) Plots representing the wide distribution of intrinsic CNNs properties. (B) Membrane resistance versus (vs) capacitance. (C) Spike half-width vs capacitance. (D) Firing frequency versus capacitance. (E) Length membrane time constant vs membrane resistance. (F) Spike half-width vs membrane resistance. (G) Short rebound vs long rebound. Values on the x-axis are the averages of firing frequencies during the first 50 ms after current offset (rebound) divided by the last 100 ms before current onset (baseline). Similarly, values on the y-axis are calculated by dividing the firing rate during the first 100 ms (blue) or 250 ms (green) after current offset by the spike rate during the last 100 ms before the current onset. Therefore, values above 1 indicate post-inhibitory rebound, while values lower than 1 indicate a decreased firing rate after the inhibition. The color coding in B-F corresponds to the neurons presented in Fig 1A (different colours of purple; dots are also encircled), to the neurons presented in Fig 2B (red, black and blue neuron; crosses are encircled), and finally all morphologically identified neurons presented in Fig 3 (the colours of the dots have the same colour as the reconstructions of the neurons). Crosses indicate the values of light-activated neurons.

We recorded *in vivo* from 144 CNNs using the whole-cell approach. Most of our recordings were targeted at the interposed nucleus, but cells were distributed across the entire medio-lateral and dorso-ventral axis (1577.2–2400 μm neuronal-depth) of the CN. As described for the whole-brain preparation and *in vitro* approach [52–56], these CNNs showed a depolarized membrane potential, generating spikes constantly (average membrane potential corrected for junction potential -40,59 ± 6,81 mV (SD)) and a strong hyperpolarization-activated depolarizing current, which in various cases gave rise to a rebound burst of action potentials (Fig 1A and 1G). We found regularly firing (N = 88) and spontaneously bursting neurons (N = 56). Of the regularly firing neurons 33 had a depolarizing after-potential (DAP) after spontaneous and evoked action potentials and 19 showed spike-frequency accommodation, whereas 41 of the bursting neurons had a DAP and 14 showed spike-frequency accommodation (Fig 1A). Regression analysis revealed that deeper neurons had a significant tendency for a lower firing frequency (R^2^ = 0.09, P = 0.001). However, all cellular electrophysiological properties of CNNs appeared to be distributed in a continuous fashion (Fig 1B–1G).

**Fig 2:**
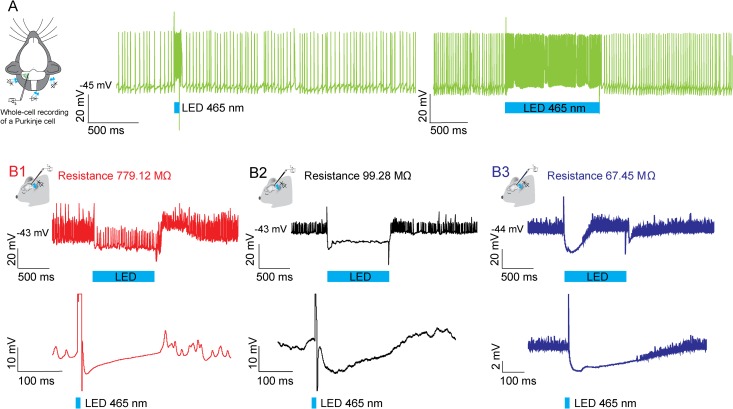
Evoked CNN activity in response to optical stimulation of PCs in L7-ChR2(H134R)-eYFP transgenic mice. The schemes represent the experimental set-up. We recorded from a neuron *in vivo*, while we shined with three blue 465 nm LED lights (indicated with blue stripes) from the outside of the brain on (A) PCs to stimulate their activity (green traces). (B) The graphs show responses of 3 CNNs to 1 second (top traces) and 10 ms (bottom traces) light stimulation (indicated with blue stripes) recorded at current clamp, illustrating the diversity in responses. Neurons with a comparable resistance (B2 and B3) can show differences in the time to reach steady state after inhibition as well as in their decay time.

**Fig 3:**
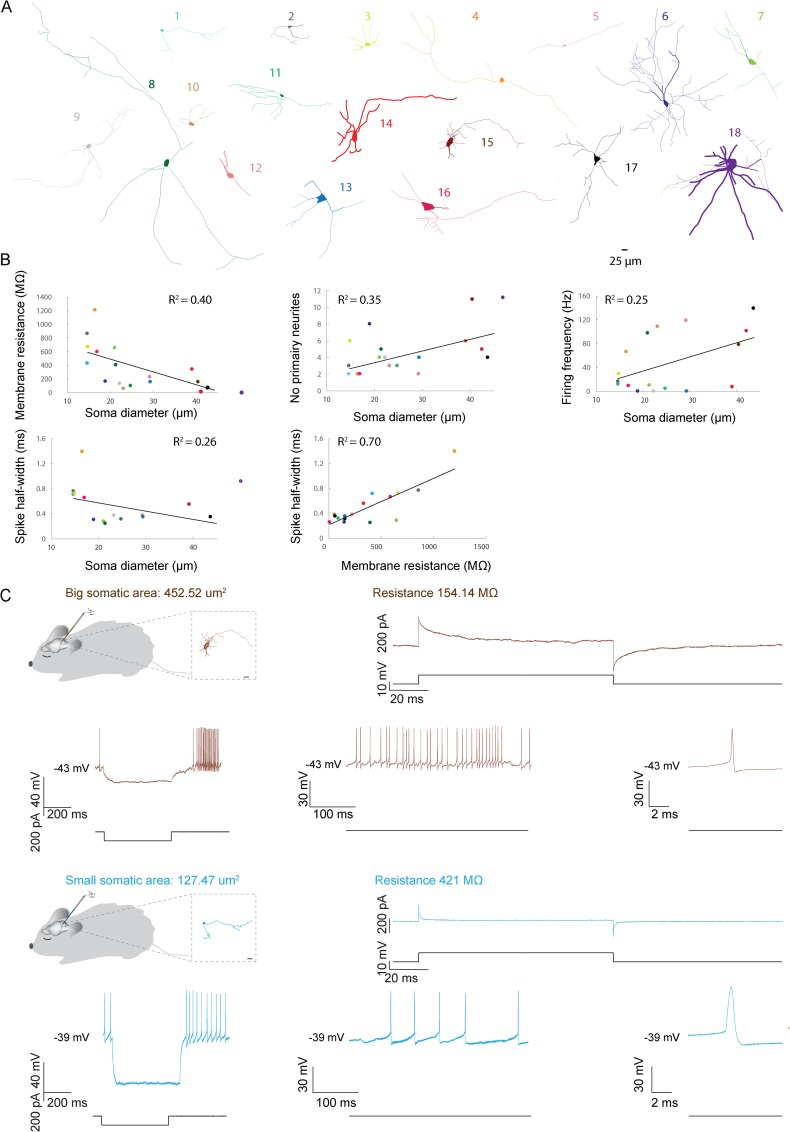
Properties of morphologically and physiologically identified CNNs. (A) Neurolucida reconstructions of cells. The scale bar (25 μm) applies to all reconstructions and is indicated at the bottom right. (B) Relation between somatic area and several morphological and electrophysiological measures. (C) Morphology and physiology of one representative large and one small CNN. Top left: schematic drawing of the experimental set-up with the morphology of the recorded neuron on the right (scale bar 25 μm). Top right: Voltage clamp recordings of the two CNNs *in vivo* in whole-cell mode. The average current response (top trace) of the corresponding neuron to ten 10 mV pulses (lower trace). Bottom graphs: Current clamp recordings of the two CNNs *in vivo* in whole-cell mode. Bottom left graphs: Top trace presents the voltage response of the reconstructed neuron to -100 pA current injection (lower trace). These traces demonstrate the I_H_ and postinhibitory rebound firing for the corresponding neuron. The middle graphs show spontaneous activity of those CNNs. The right traces show the peak-aligned average of the spikes, highlighting the differences in the spike half-width comparing small and large neurons.

To find out whether CNNs could be identified by occupying unique territories in the space of particular parameters, we first measured the input resistance, capacitance, membrane time constant, spike width at half amplitude (half-width), firing frequency and regularity, each of which may contribute to the identity of a CNN [11,25,57]. Parameter values varied over more than one or two orders of magnitudes for input resistance (9.63–3352.1 MΩ), capacitance (6.7 to 772.57 pF), membrane potential (-61.85 –-18.98 mV), membrane time-constant (0.23–21.7 ms), firing frequency (0–176.6 Hz), regularity of firing (0.09–9.23) and spike half-width (0.178–1.98 ms) (see also Fig 1B–1F). Capacitance decreased with membrane-resistance (R^2^ = 0.12, P<0.001) and spike half-width (R^2^ = 0.27, P<0.001), while capacitance increased with firing frequency (R^2^ = 0.29, P<0.001). The membrane resistance decreased with membrane time constant and increased with spike half-width (R^2^ = 0.045, P = 0.031 and R^2^ = 0.023, P<0.001, respectively), while no statistically significant linear dependence of membrane resistance and regularity of firing (R^2^ = 0.033, P = 0.055) or overall firing frequency (R^2^ = 0.030, P = 0.076) was detected. In a next step we further quantified the voltage firing threshold (-28 –-49 mV), the maximum firing frequency (10–202 Hz) in response to a 100 pA step, the amplitude of both spontaneous (18–80 mV) and evoked action potentials (17–76 mV), and the amplitude (0–18 mV) and time point of the fast after-hyperpolarization peak (fAHP) following a spike (0,2–3,8 ms). The spike amplitude increased with membrane resistance (spike amplitude: correlation resistance R^2^ = 0.052, P = 0.018, and capacitance R^2^ = 0.001, P = 0.768) and the time to peak of the fAHP decreased with increasing capacitance (time fAHP: correlation resistance R^2^ = 0.003, P = 0.594, and capacitance: R^2^ = 0.087and, P = 0.003). All other parameters did not correlate with membrane resistance and capacitance (Voltage firing threshold: correlation resistance R^2^ = 0.030, P = 0.068, and capacitance R^2^ = 0.017, P = 0.194; maximum firing frequency: correlation resistance R^2^ = 0.041, P = 0.135, and capacitance R^2^ = 0.002, P = 0.762; evoked spike amplitude: correlation resistance R^2^ = 0.038, P = 0.236, and capacitance R^2^ = 0.005, P = 0.687; amplitude fAHP: correlation resistance R^2^ = 0.001, P = 0.846, and capacitance R^2^ = 0.031, P = 0.082).

We next investigated post-inhibitory rebound spiking following hyperpolarizing current injections into the soma. We calculated the increase in firing frequency 50, 100 and 250 ms after current offset compared to 100 ms before current onset (Fig 1G), because previous work revealed differences in short and long bursts of rebound firing in CNNs [58–61]. When analyzing the firing rate increase over 50 ms versus 100 and 250 ms, many neurons showed short or long rebounds [58,61–63]. Neurons with a higher resistance showed a significant trend for shorter and more intense rebounds compared to neurons with a lower resistance (50 ms rebound R^2^ = 0.103, P = 0.011; 100 ms rebound R^2^ = 0.1503, P = 0.0011; and 250 ms rebound R^2^ = 0.048, P = 0.068).

To find out whether we could identify specific clusters of neurons by combining the analyses of different previously suggested parameters [25,57,64], namely input resistance, membrane time constant, firing rate, CV value, CV_2_ value, spike half-width and action potential amplitude, we employed k-means clustering. We employed several strategies to identify clusters of neurons. Clusters based on single cell physiological parameters explained well over 80% of variance for four clusters of neurons. The clusters obtained this way however were in poor agreement when clustering was performed with different parameters. In a second strategy we clustered based on one parameter and added more parameters to obtain better agreement between clustering obtained with different input parameters. Although clusters agreed more in these cases, the variance explained dropped dramatically when adding parameters, with less than 30% variance explained when using two input parameters. These results indicate that the neuronal population of the CN is highly varied and that cell physiological parameters have a tendency to vary irrespective of other cell physiological parameters.

When using k-means clustering on firing frequency, spike half-width and resistance, as employed previously [25,57,64], there was little agreement between the cluster outcomes when comparing between different input parameters for the clustering algorithm. Manual clustering using spike half-widths reported in literature (using a Q10 value of 1.86 as described by [25]; but see also [57] and Table 1) resulted in three clusters for each set of values. Although these clusters were significantly different from each other on some parameters, the separations were not consistent on all parameters (i.e. membrane resistance and capacitance clustered with spike half-width in whole-cell mode, but firing frequencies in whole-cell and cell-attached mode did not). Spike amplitude and the time to peak of the fAHP did also not allow clustering of neurons. We conclude that the various physiological parameters of CNNs show specific and significant relations with each other, but without additional characteristics they hold relatively little information on their identity.

**Table 1:** Cerebellar Nuclear Neurons split up by spike half-width (HW).

Borders	N	Membrane resistance (MΩ)	Membrane capacitance (pF)	Spike half-width spontaneous spikes (ms)	Frequency attached (Hz)	Frequency whole-cell (Hz)
HW<0.42	50	144.3 ± 105.8 (220)	205.3 ± 137.9 (141.7)	0.31 ± 0.06 (0.74)	28.3 ± 26.1 (16.9)	56.3 ± 35.3 (30.6)
0.42<HW <0.60	24	242.4 ± 138.9 (572)	184.8 ± 82.1 (69.2)	0.48 ± 0.05 (1.15)	34.0 ± 26.5 (8.3)	46.1 ± 22.7 (28.5)
0.60<HW	29	742.5 ± 814.4 (1,069)	61.6 ± 35.1 (55.9)	0.89 ± 0.24 (1.52)	17.1 ± 14.7 (9.8)	29.2 ± 22.9 (9.9)
		**1 vs 2 *P*<0.01** 2 vs 3 *P*>0.05 **1 vs 3 *P*<0.01**	1 vs 2 *P*>0.05 **2 vs 3 *P*<0.01** **1 vs 3 *P*<0.01**	**1 vs 2 *P*<0.01** **2 vs 3 *P*<0.01** **1 vs 3 *P*<0.01**	1 vs 2 *P*>0.05 2 vs 3 *P*>0.05 1 vs 3 *P*>0.05	1 vs 2 *P*>0.05 2 vs 3 *P*>0.05 **1 vs 3 *P*<0.01**
HW<0.85	89	255.8 ± 332.6	176.0 ± 122.0	0.43 ± 0.17 (0.49)	28.3 ± 25.1	48.6 ± 31.4 (22.2)
0.42<HW <0.9	42	424.2 ± 466.8 (31)	131.7 ± 84.5 (229)	0.60 ± 0.16 (0.61)	27.3 ± 23.9	37.7 ± 21.9 (48.6)
0.9<HW	11	867.0 ± 1079.7	45.0 ± 36.1	1.10 ± 0.26 (1.1)	18.9 ± 18.3	33.8 ± 32.2 (9.6)
		1 vs 2 *P*>0.05 2 vs 3 *P*>0.05 1 vs 3 *P*>0.05	**1 vs 2 P<0.01** **2 vs 3 *P*<0.01** **1 vs 3 *P*<0.01**	1 vs 2 *P*>0.05 **2 vs 3 *P*<0.01** **1 vs 3 *P*<0.01**	1 vs 2 *P*>0.05 2 vs 3 *P*>0.05 1 vs 3 *P*>0.05	1 vs 2 *P*>0.05 2 vs 3 *P*>0.05 1 vs 3 *P*>0.05

Comparison of our CNN recordings to those done by Uusisaari et al. (2007) [25] (top four rows) and by Bengtsson et al. (2011) [59] (bottom four rows). Our CNN recordings are split up to match the presentation in the published observations. Data from our dataset are given as means ± SD. When available, the data published by our colleagues are indicated in parenthesis directly below our data. Spike half-widths were calculated for 37°C with a Q10 value of 1.86 via HW_37_ = HW_24_/(1.86)^((37°C– 24°C)/10)). Significance is indicated below each column (t-test with Bonferroni correction).

### Integration of Purkinje cell inhibition

Next we were interested whether optical stimulation of GABAergic PCs *in vivo* allowed for differentiation of CNN subtypes in line with what has been found *in vitro* [65]. Therefore, we inhibited 16 CNNs for 1 second and 7 CNNs for 10 ms with strong optical stimulation of PCs in the *in vivo* whole-cell preparation [37] (Fig 2). By crossing the channelrhodopsin 2 (ChR2, Ai32 line) with the L7-cre line (for references see methods section), the fusion protein channelrhodopsin-EYFP was exclusively expressed in PCs. There was no significant expression in neurons in the forebrain or brainstem. Other neurons in the cerebellum also did not show the fusion protein (see also [37], but see [66]). Three blue LED lights were positioned around the cerebellum of the animal. The LED lights were driven by a linear LED driver. When the light stimulus was turned on for 10 ms or 1 second, PCs were depolarized, which evoked an increase in the simple spike firing frequency from 72 ± 19 Hz to 117 ± 30 Hz (N = 7 recorded PCs, Fig 2A). We recorded from PCs up to a depth of 1575 μm throughout the cortex, which indicates that the vast majority of cerebellar PCs were activated by the light. The firing frequency of CNNs decreased with the strength of stimulation [37]. Strong PC light stimulation evoked a reduction in firing rate up to silencing of CNNs (baseline firing frequency CNNs: 68 ± 11 Hz, firing frequency during 1 second stimulation: 12 ± 5 Hz, Fig 2B). Recorded CNNs were well distributed over the entire parameter space observed in our previous experiments (membrane resistance 11.7–779.1 MΩ; capacitance 110.17–664.88 pF; membrane potential -44.95 –-24.85 mV; firing frequency 1–183 Hz; membrane time constant 0.11–3.12 ms; spike half-width 0.18–0.45 ms; see also Fig 1B–1F; all properties F-test not significant). Membrane resistance did neither have a predictive value for the decay time (DT) nor for the time it takes the membrane to reach the steady state membrane potential (SS) (after 1 s stimulation: correlation resistance—DT R^2^ = 0.144, P = 0.278, and resistance—SS R^2^ = 0.17, P = 0.100; after 10 ms stimulation: correlation resistance—DT R^2^ = 0.023, P = 0.775, and resistance—SS R^2^ = 0.058, P = 0.565). Additionally, we did not find a relation between membrane resistance and the duration and amplitude of the evoked inhibition (after 1 s stimulation: correlation resistance–duration R^2^ = 0.047, P = 0.415, and resistance—amplitude R^2^ = 0.018, P = 0.621; after 10 ms stimulation: correlation resistance—duration R^2^ = 0.000, p = 0.996, and resistance—amplitude R^2^ = 0.093, P = 0.503). These data indicate that optical stimulation of PCs in the whole-cell mode in the anesthetized preparation by itself does not allow a clear separation of CNNs.

### Identification of CNNs using a combination of physiological and morphological properties

To find out whether there is an association between neuronal morphology and physiological cell identity in the CN we labelled 18 of the 144 CNNs in 18 mice with Neurobiotin during whole-cell recordings *in vivo*; seventeen of these cells were located in the interposed nucleus (N = 17), while 1 cell was localized in the medial nucleus (N = 1). All cells were reconstructed and the diameter of the cell body and their dendritic tree could be well discerned (Fig 3A). The soma size of the individual neurons showed a large variation with the soma diameter varying between 14.7 and 50 μm and soma area between 56.85 and 902.72 μm^2^ (Table 2). Similarly to filled neurons studied *in vitro* [25], the 5 smallest neurons showed a less complex dendritic morphology compared to the larger ones (Fig 3A). The diameter of the soma and the area had a predictive value for the number of primary neurites (R^2^ = 0.35, P = 0.009 and R^2^ = 0.37, P = 0.007; Fig 3B). Both neuron 6 and neuron 9 formed special cases; neuron 6 had a small cell body, 8 primary neurites and a complex tree of primary and secondary neurites, while neuron 9 provided an axon to the granule cell layer of the copula pyramidis [14,15,27]. Possibly these neurons represent glycinergic non-spiking CNNs [11–13]. Overall, the spatial distribution of neurites, the amount of primary neurites and their total length varied as a continuum (Fig 3A).

**Table 2:** Morphological properties of reconstructed CNNs.

		Soma			Neurites	
Cell number	Diameter (μm)	Area (μm^2^)	Aspect Ratio	Roundness	Primary neurites	Total length reconstructed (μm)
**1**	14.7	127.47	1.2307	0.7548	2	746.3
**2**	14.8	62.2093	2.0379	0.4412	3	361.8
**3**	14.9	94.2	1.682	0.521	6	357.1
**4**	16.5	140	1.65	0.6513	2	975.3
**5**	17.0	56.85	3.237	0.2492	2	514.4
**6**	19.0	128.45	1.6198	0.4516	8	2894.9
**7**	21.1	189.61	1.3754	0.5428	4	635.2
**8**	21.4	195.46	1.729	0.5812	5	2464.2
**9**	22.3	302	1.2813	0.7743	4	1649.5
**10**	23.2	265.67	1.537	0.6301	3	453.6
**11**	24.8	122.86	1.7757	0.5452	3	1552.8
**12**	29.3	493.26	2.0379	0.6579	2	258.8
**13**	29.4	347.711	1.4651	0.5125	4	580.4
**14**	39.1	375.27	2.1617	0.3818	6	1294.8
**15**	40.5	452.52	2.2326	0.3748	12	1208.8
**16**	42.2	634.79	1.9531	0.4529	5	1019.1
**17**	43.7	458.49	1.8715	0.306	4	1896
**18**	50.0	902.72	1.9148	0.4603	12	3570.4

Soma size and statistics on neurites of CNNs

Regarding physiological properties, these neurons were well distributed over the entire parameter space observed in our previous experiments (variance of membrane resistance: 9.6–1204.99 MΩ, membrane capacitance 7.63–354.93 pF, firing frequency 4.41–138.38 Hz, and spike half-width 0.24–1.39 ms; for illustrative purposes see also color-coded dots in Fig 1B–1F, which are the same color-codes used in Fig 3). There was no statistically significant difference in variance between the neurons that were morphologically identified and those that were not (all properties F-test not significant). With the exception of neurons 6, 9 and 13, all morphologically identified CNNs showed intrinsic activity during cell-attached and intracellular recordings. Frequencies varied from 0 to 138.38 Hz (Fig 3B). As described above for the overall population of CNNs recorded in this study, membrane resistance decreased and capacitance increased with soma diameter, soma perimeter and area (Resistance: diameter R^2^ = 0.40, P = 0.004, perimeter R^2^ = 0.44, P = 0.002, area R^2^ = 0.32, P = 0.01; capacitance: diameter R^2^ = 0.28, P = 0.027, perimeter R^2^ = 0.30, P = 0.024, area R^2^ = 0.14, P = 0.014, Fig 3B). The diameter of the soma and the area had a predictive value for firing frequency (R^2^ = 0.25, P = 0.03 and R^2^ = 0.39, P = 0.005) and spike half-width (R^2^ = 0.26, P = 0.035 but R^2^ = 0.20, P = 0.07; Fig 3B). Within the group of morphologically identified neurons we found that membrane-resistance increased with spike half-width (R^2^ = 0.70, P>0.0001), whereas membrane resistance did not have a predictive value for the length of the membrane time constant (R^2^ = 0.02, P = 0.065) or firing frequency (R^2^ = 0.20, P = 0.075). Similarly, resistance, area, and diameter of the soma did not have a predictive value for the regularity of firing (R^2^ = 0.01, P = 0.68; R^2^ = 0.02, P = 0.55; R^2^ = 0.141, P = 0.134, respectively). In contrast to previous findings [67], we were not able to detect any significant difference between recordings with and without Neurobiotin in any of the cell populations.

When we grouped the 40% largest (29.3–50 μm in diameter) and 40% smallest (14.7–21.1 μm in diameter) CNNs in line with former *in vitro* studies [17], we found significant differences in soma area (df = 7; P>0.001), minimum soma diameter (df = 12, P>0.001) and perimeter (df = 7, P>0.001) (Table 2). Yet, interestingly, the roundness of the soma (df = 11, P = 0.194) and the aspect ratio (i.e. minimal somatic diameter / maximal somatic diameter; df = 9, P = 0.36) did not differ between larger and smaller neurons. When relating morphological characteristics to the cell physiological properties, we were able to discriminate the cell physiological characteristics of the larger and smaller cells with a 95% confidence interval. In line with former *in vitro* studies on CNNs, which indicated that spike half-width and membrane resistance are amongst the most informative parameters [25,57,64], we found that smaller CNNs had a significantly larger spike half-width (df = 8, P = 0.028) and bigger input resistance (df = 7, P = 0.003) compared to larger neurons (Fig 3B and 3C). Smaller CNNs also tended to have a lower capacitance compared to larger neurons, but this difference did not reach significance in our limited dataset (df = 7, P = 0.080). We did not find any significance or trend when comparing firing frequencies and CV values of all the larger neurons with those of all the smaller neurons (df = 10, P = 0.071 and df = 10, P = 0.235, respectively). However, when excluding the three non-spiking neurons (CNN 6, 9 and 13), which had an intermediate resistance and capacitance, the analysis revealed a slight but significant difference in firing frequency (df = 9, P = 0.048). Finally, analysis of the post-inhibitory rebound over the 50 ms, 100 ms and 250 ms periods following hyperpolarizing current injections into the soma also did not reveal any difference between the small and larger neurons (50 ms P = 0.245, 100 ms P = 0.348, 250 ms P = 0.262).

## Discussion

We performed whole-cell current-clamp recordings from 144 individual CNNs in ketamine-xylazine anesthetized mice *in vivo*. We predominantly, but not exclusively, targeted the interposed nucleus. The neurons were allocated to different depths within the CN and a subset of 18 random neurons across the CN was reconstructed at the morphological level. Seventeen of 18 CNNs were located in the interposed nucleus, while one was localized in the medial nucleus. Given the limitations of our approach, we could not discern any apparent correlation between the location of the recordings and the cell physiological properties. We assessed whether the specific CNN classes that have been described for young animals *in vitro* [12,23–27] could also be identified *in vivo* in adult animals. Since recordings *in vitro* are likely to show less variation in physiological properties compared to the *in vivo* situation [32], we expected to obtain additional information on the cellular properties of CNNs, allowing fine-tuning of local computational CN network models and scale computational modeling networks of the olivocerebellar system as a whole [40–43]. Our recordings point towards several main interpretations that may further elucidate the underpinnings of cerebellar motor coordination [30, 34–37]. In general, most cell physiological parameters varied more than one order of magnitude without significant correlations with one another, suggesting that the complex CN functionality is assisted by a rich repertoire of neuronal properties that encode and integrate cerebellar cortical information in a cell specific manner without prevalence of particular classes of cells. Indeed, using k-means clustering based on the cell physiological parameters alone we could not identify specific clusters of CNNs, which precludes online classification of CNN cell types in the anesthetized preparation *in vivo* purely based on intrinsic physiological properties. Moreover, unlike the *in vitro* situation [65], the integrative properties of CNNs such as time constant of IPSCs or rebound following PC activation also formed a continuum and also did not show uniquely defined types of CNNs, when merely analyzed with electrophysiology. However, the capacitance of CNNs *in vivo* decreased with membrane-resistance and neurons with a higher resistance showed a significant trend for shorter and more intense rebounds compared to neurons with a lower resistance. Moreover, in line with the general correlations between capacitance and cell surface area in neurons [68], we could distinguish the electrophysiological properties of larger neurons (24.7–50 μm in diameter) from those of the smaller CNNs (< 21.1 μm in diameter), with the larger cells showing lower membrane-resistance and a smaller spike half-width. Thus, general cell physiological parameters like membrane resistance, membrane time constant, spike half-width and firing frequency of CNNs could not be clustered in the anesthetized *in vivo* preparation revealing specific classes of cells, but soma size mattered as revealed by the post hoc morphological analysis.

### Large variation of cell physiological parameters within cerebellar nuclei

Most of the in vivo electrophysiological parameters varied more than one order of magnitude with a parameter space bigger than that of most *in vitro* data sets of the cerebellar and vestibular nuclei [54,65,69,70]. Therefore, it should be noted that the variability in recording quality of whole-cell recordings might potentially also influence the statistical outcome. Such a big variation in parameters is also exceptional compared to neurons in cortical brain areas such as the entorhinal cortex [33], barrel cortex [34], prefrontal cortex [35], motor cortex [36], and even the cerebellar cortex itself [37,39]. In most cortical regions variations in cell physiological parameters such as membrane resistance, membrane time constant and firing rate are smaller compared to those in CNNs, which underlines the complex functionality and integrative properties of the CN [11,17,22,25]. This high level of variability in cellular properties may also reflect the differences in functionality between the different subdivisions of the CN [71]. Lateral nuclei receive most of their PC inputs from cerebellar hemispheres and send their output to the (pre)motor cortex via the thalamus, possibly contributing to behavioural planning; the interposed nucleus receives most of its inputs from the paravermis and plays an important role in more simple forms of conditioning; and finally, the fastigial or medial cerebellar nuclei receive their main input from the floccunodular lobe and vermis, facilitating balance and oculomotor functions [71]. In this respect the CN somewhat resemble the CA1 area of the hippocampus, which integrates inputs from the medial and lateral entorhinal cortex and which also does not allow cellular identification based on electrophysiological parameters alone [72–74]. Despite the enormous ranges in parameter space of CNNs, we observed a continuum of values for all cell physiological parameters, including the occurrence of rebound. Given that the duration of rebounds of CNNs probably depends predominantly on the types of ion conductances on their cell membrane [59,60] and the activity of their afferent inputs [75], we also tested correlations between the types of rebound and the other cell physiological parameters. However, despite the substantial variety in rebounds [58–61], these correlations were also not sufficiently informative for identifying specific subtypes of CNNs. This continuous distribution of cell parameters is however in accordance with the previous *in vitro* experiments on the cerebellar and vestibular nuclei, which also showed a widespread overlap in physiological parameters of individual neuronal subtypes [25,54,69,70,76]. Thus, since cell physiological properties varied irrespective of other physiological parameters, these data suggest that CN encode and integrate cerebellar cortical information in a specific manner at the single cell level and endows the network with the capacity to perform complex cell and area specific biological processes.

To improve the sensitivity of our analyses of CNN cell physiological parameters, one could in future studies consider to further decrease the variability in the data set with regard to location. Indeed, the regression analysis of the predictive value of neuronal-depth for the firing frequency was significant, which suggests a relation between the neuronal depth and the frequency of firing due to differences in input, output or intrinsic neuronal activity along the anterior-posterior axis. Since all CNNs receive region-specific inputs from the cerebellar cortex assigned to a limited set of functions, a sufficient amount of recordings from neurons from a particular cerebellar nucleus at a certain depth and medial-lateral axis would likely decrease the overall variability, and thereby enhance the chances for physiological identification. However, focusing purely on one nucleus would decrease the generalized impact of the findings.

### Size matters in structure—function relation

In line with previous reports describing the morphology of CNNs, we were not able to discriminate between neuron groups solely based on their morphology [1,69,77]. Next to a pure morphological staining one needs to at least study the neurotransmitter expression pattern to be able to discriminate between glutamatergic, GABAergic and glycinergic neurons *in vivo* [11,22,26]. In addition, it will probably be helpful if one could define the efferent projections to study the connectivity of the individual CNN with its network [7,8,11–13,17,20,21,72]. Similar to previous *in vitro* results we found that smaller neurons have a less complex dendritic morphology [28], which suggests that smaller neurons integrate information differently compared to larger neurons with a more complex widespread dendritic tree.

Combining morphological with whole-cell physiological parameters revealed various trends, allowing physiological identification of larger and smaller cells with 95% confidence intervals. Compared to the smaller cells, larger cells had a lower membrane resistance, with a tendency for higher capacitance. It is tempting to speculate that these smaller neurons are GABAergic, because it has been suggested that glutamic acid decarboxylase (GAD) positive neurons are smaller compared to glutamatergic neurons and also have a longer spike half-width [13,25]. The larger neurons are most likely the glutamatergic CNNs [25]. *In vitro* the larger neurons can be subdivided into two subpopulations, namely small and large GAD negative neurons [25]. Larger neurons have a higher capacitance with a shorter spike half-width and no spike frequency adaptation. Smaller neurons have a lower capacitance with a broader action potential, a strong late component after-hyperpolarization and longer rebound (for review see [17]). Our whole-cell *in vivo* recordings of post-hoc morphologically identified cells under anesthesia support these trends and will allow modelers of the CN to further expand and fine-tune the computational repertoire of CNNs in intact animals [63,78,79].

## Abbreviations

ChR2: Channelrhodopsin 2
Cm: Membrane capacitance
CN: Cerebellar nuclei
CNNs: Cerebellar nuclei neurons
CV: Coefficient of variation
DAB: 3,3′-Diaminobenzidine
DAP: Depolarizing after-potential
DT: Decay time
eYFP: Enhanced yellow fluorescent protein
fAHP: Fast after-hyperpolarization peak
GABA: Gamma-aminobutyric acid
GAD: Glutamic acid decarboxylase
HW: Half-width
i.e.: Id est
ISI: Inter-spike-intervals
LED: Light-emitted diode
PB: Phosphate buffer
PB-TX: Phosphate-Triton buffer
PCs: Purkinje cells
Ra: Access resistance
Rm: Membrane resistance
SD: Standard deviation
SS: Steady state
vs: Versus
